# Calorimetry of a Bose–Einstein-condensed photon gas

**DOI:** 10.1038/ncomms11340

**Published:** 2016-04-19

**Authors:** Tobias Damm, Julian Schmitt, Qi Liang, David Dung, Frank Vewinger, Martin Weitz, Jan Klaers

**Affiliations:** 1 Institut für Angewandte Physik, Atominstitut, Institute of Quantum Electronics, Universität Bonn, Wegelerstrasse 8, 53115 Bonn, Germany

## Abstract

Phase transitions, as the condensation of a gas to a liquid, are often revealed by a discontinuous behaviour of thermodynamic quantities. For liquid helium, for example, a divergence of the specific heat signals the transition from the normal fluid to the superfluid state. Apart from liquid helium, determining the specific heat of a Bose gas has proven to be a challenging task, for example, for ultracold atomic Bose gases. Here we examine the thermodynamic behaviour of a trapped two-dimensional photon gas, a system that allows us to spectroscopically determine the specific heat and the entropy of a nearly ideal Bose gas from the classical high temperature to the Bose-condensed quantum regime. The critical behaviour at the phase transition is clearly revealed by a cusp singularity of the specific heat. Regarded as a test of quantum statistical mechanics, our results demonstrate a quantitative agreement with its predictions at the microscopic level.

## Abstract (web summary)

Phase transitions are often revealed by a discontinuous behaviour of thermodynamic quantities. Here, the authors study the thermodynamic behaviour of a trapped 2D photon gas, revealing critical behaviour at the phase transition through a cusp singularity of the specific heat.

## Graphical abstract

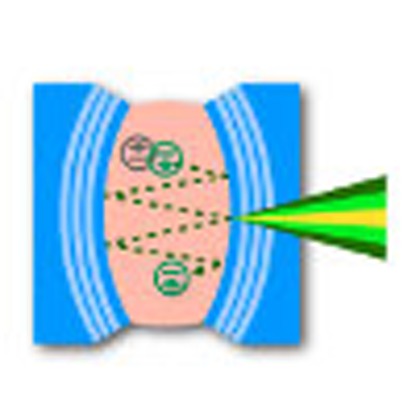

Below 2.2 K, liquid helium shows peculiar hydrodynamic properties, such as a flow without viscosity, the fountain effect or the formation of vortices^1^. This transition from a normal fluid to a superfluid has been named *λ*-transition, which originates from the fact that plotting the heat capacity versus temperature^2^ results in a graph resembling the greek letter *λ*. Soon after this discovery, it has been proposed that superfluid helium forms a macroscopic matter wave as a consequence of Bose–Einstein condensation^3^, which describes the condensation of the ideal (interaction-free) Bose gas at low temperatures due to quantum statistics^4^. This idea proved to be fruitful despite the fact that liquid helium is far from a system of interaction-free particles^5^. The impressive progress in the cooling of dilute atomic gases has paved the way to realize weakly interacting Bose gases at nano-Kelvin temperatures^6^,^7^. Here the relation to Bose–Einstein condensation has been immediately clear. Interestingly, in contrast to liquid helium and a recent measurement of a strongly interacting atomic Fermi gas^8^, these systems have not allowed for detailed calorimetric studies up to now^9^. Evidence for a non-classical specific heat has been reported^10^,^11^, but the accuracy obtained in experiments with weakly interacting atomic Bose gases has not been sufficient for an unambiguous determination of the temperature dependence of the heat capacity.

Following ultracold atomic Bose gases, other physical systems have been demonstrated to undergo Bose–Einstein condensation, for example, gases of exciton–polaritons^12^,^13^, magnons^14^ and, in previous work of our group, photons^15^,^16^,^17^. In contrast to a three-dimensional thermal photon gas as Planck's blackbody radiation, photons can exhibit Bose–Einstein condensation, if the thermalization process is restricted to two motional degrees of freedom. Experimentally, this situation has been realized in a microcavity enclosing a dye medium, designated as a room temperature heat bath for the photon gas. Detailed experimental studies of the thermalization^16^ and condensation process^15^, as well as the quantum statistics of the photon condensate^17^, have revealed the signatures of an almost ideal Bose gas.

Here we report a measurement of the calorimetric properties of a Bose–Einstein-condensed photon gas, in particular, the temperature dependence of the specific heat and entropy from the classical high temperature to the quantum-degenerate regime at low temperatures. At the phase transition, the observed specific heat shows a cusp singularity, illustrating critical behaviour for a photon gas analogous to the *λ*-transition of liquid helium.

## Results

### Two-dimensional photon gas in a dye microcavity

**Figure 1:**
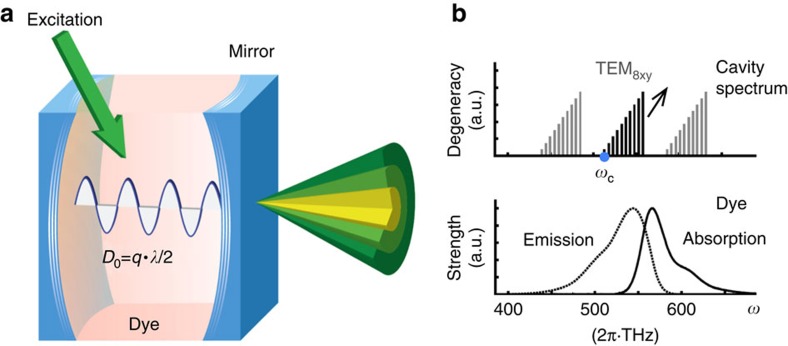
Bose–Einstein condensation of a two-dimensional photon gas. (**a**) Photons are captured inside a microcavity consisting of two spherically curved mirrors and get repeatedly absorbed and re-emitted by the embedded dye medium, leading to a thermalization of the photon gas to the temperature of the resonator (room temperature). (**b**) The short cavity length causes a large frequency gap between the longitudinal resonator modes (free spectral range) of order of the emission bandwidth of the dye molecules. In this situation, the resonator becomes populated by photons of a single longitudinal mode number only, here *q*=8. However, the photons may still populate a multitude of transversally excited cavity modes (TEM_8xy_ sub-spectrum), which effectively makes the photon gas two-dimensional. Above a critical photon number, the photon gas undergoes a Bose–Einstein condensation, leading to a massive population of the cavity ground mode (TEM_800_). Thermodynamic information is obtained by spectroscopically analysing the photon energy distribution across the phase transition.

In our experiment (Fig. 1a), photons are captured inside a microcavity consisting of two spherically curved mirrors while repeatedly being absorbed and re-emitted by the embedded dye medium. The cavity length is of the same order as the wavelength itself, which causes a large frequency gap between the longitudinal resonator modes (free spectral range), comparable to the emission bandwidth of the dye molecules (Fig. 1b). In this situation, the resonator becomes populated by photons of a single longitudinal mode number *q* only, for example, *q*=8. While the longitudinal mode number is frozen out, the photons may populate a multitude of transversally excited cavity modes, for example, the TEM_8xy_ sub-spectrum, which makes the photon gas effectively two-dimensional. The photon energy-momentum-relation acquires a quadratic form, resembling that of a massive particle, and a trapping potential for the photon gas is induced by the mirror curvature. One can show that the photon gas confined in the resonator is formally equivalent to a harmonically trapped two-dimensional gas of massive bosons^18^,^19^, described by the dispersion


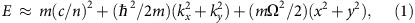


with spatial coordinates *x* and *y*, transverse wave vector components *k*_*x*_ and *k*_*y*_, trapping frequency Ω and an effective mass *m*=*ħω*_c_(*n*/*c*)^2^, where *ħω*_c_ is the photon energy in the cavity ground mode with *n* as the refractive index of the medium and *c* as the vacuum speed of light. Thermal equilibrium of the photon gas with the cavity environment at room temperature is achieved via repeated absorption and emission processes by the dye molecules, which establishes a thermal contact between photon gas and optical medium^18^,^20^,^21^. Other than in a blackbody radiator, the thermalization process allows for an independent adjustment of temperature and photon number, for example, by (initial) optical pumping, which eventually goes back to a separation of energy scales of photon energy and thermal energy. In our experiment, the Bose–Einstein condensation is triggered by increasing the photon number above the saturation level at a given temperature. The corresponding critical particle number is given by^22^,^23^,^24^


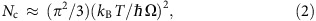


which for typical experimental parameters corresponds to *N*_c_≈90,000.

One of the benefits of the given experimental system is that one can easily interpolate between equilibrium and non-equilibrium experimental conditions. Parameters, such as mirror reflectivity, dye concentration, cavity tuning and pump geometry, can be chosen such that gain and dissipation either significantly contribute to the system dynamics or effectively drop out of it^16^,^20^,^25^,^26^,^27^. In this study, we have concentrated on the equilibrium properties of the system. Details on the corresponding experimental parameters can be found in the Methods section. In our experiments, the optical medium is pumped with a spectrally off-resonant laser source at a wavelength of *λ*_exc_=532 nm, having a relatively large beam diameter of ∼150 μm to keep the excitation of the medium nearly spatially homogeneous. Two acousto-optical modulators (AOMs) are used to chop the pump light to long pulses of 400ns length with a repetition rate of 400 Hz. The AOMs further control the intensity of each light pulse that allows us to adjust the average photon number with respect to the critical photon number. In our experiment, we quickly ramp the total photon number from typically 30,000 to 550,000 photons within 250 ms.

The thermodynamic properties of the system are experimentally accessible by spectroscopic means. To obtain the intracavity spectral distribution of the photons, we measure the spectral distribution outside the resonator and divide by the (wavelength-dependent) transmission coefficient of the mirrors. For this, the emitted cavity light is collimated and coupled to a monochromator (4*f* set-up), where it is spectrally decomposed by a diffraction grating (1,200 grooves per mm), giving an overall resolution of 0.5 nm. With this set-up, it is possible to capture the photon gas spectrum in the wavelength region starting from ≈550 nm to the cavity cutoff at *λ*_c_=580 nm corresponding to an energy range of ≈4 *k*_B_*T*. The latter comprises the condensate population and ≈95% of photons in excited cavity modes. The ≈5% most energetic photons of the thermal cloud are not experimentally resolvable with the present set-up.

**Figure 2:**
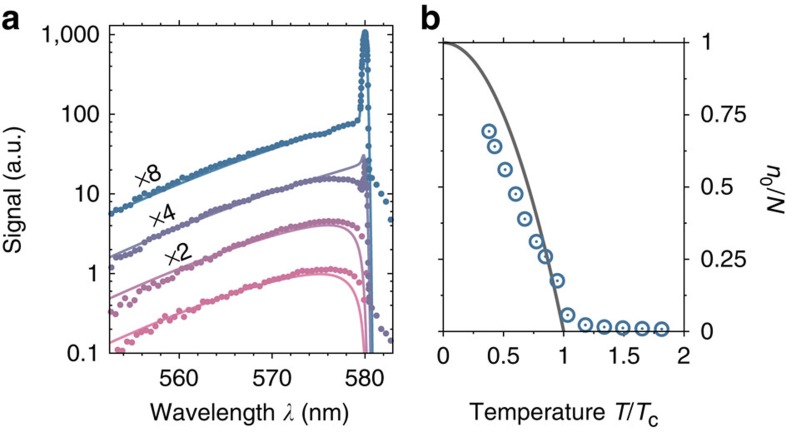
Spectral photon distribution and condensate fraction. (**a**) Distribution of photon energies for increasing total photon number (circles). For clarity, the spectra have been vertically shifted. The observed spectra agree well with the expected 300 K Bose–Einstein distribution functions (solid lines). (**b**) Condensate fraction *n*_0_/*N* versus the reduced temperature *T*/*T*_c_ along with the theoretical expectation *n*_0_/*N=*1−(*T/T*_c_)^2^. Due to an imperfect saturation of the population of the thermal cloud, the condensate fraction is observed to be systematically below the theoretically expected values.

Figure 2a shows spectra obtained at fixed temperature *T*=300 K for total photon numbers ranging from *N*≈30,000 to *N*≈550,000 (varying chemical potential). The observed spectra generally are in good agreement with Bose–Einstein distributions, with residual deviations at the lower photon energy part due to imperfect reabsorption. The measured energy distributions can be used to obtain full thermodynamic information of the two-dimensional photon gas. All of the derived quantities will be measured for constant volume, which here means constant (inverse squared) trapping frequency, and fixed absolute temperature *T*. As a first step, we have determined the condensate fraction *n*_0_/*N* as a function of the reduced temperature *T*/*T*_c_ (Fig. 2b). From equation (2) follows that the reduced temperature *T*/*T*_c_ is related to the total particle number by *T*/*T*_c_=(*N*_c_/*N*)^1/2^, with the total particle number *N* being obtained by integrating over the spectrum. The experimentally derived condensate fractions are slightly below the theoretical expectations shown by the solid line in Fig. 2b, describing an inverse parabola *n*_0_/*N*=1−(*T*/*T*_c_)^2^. This stems from an imperfect saturation of the population in excited photon modes, which has already been observed in previous measurements^15^, and potentially originates from a weak (thermo-optically induced) photon self-interaction^28^.

### Caloric and entropic properties

We next determine the average energy per photon *U*/*N*, with the zero point of the energy scale being set to the energy *ħω*_c_*=hc/λ*_c_ of the cavity ground state (TEM_00_ mode), corresponding to a condensate wavelength of *λ*_c_=580 nm. On the basis of the experimentally obtained spectral photon distribution *n*(*λ*) in the wavelength regime λ≈550–580 nm, we extrapolate the total internal energy to be *U≈κ* × ∫_550 nm_^580 nm^ *n*(*λ*) *hc* (λ^−1^−λ_c_^−1^) *dλ*, with *h* as Planck constant and *c* as the vacuum speed of light. The extrapolation factor *κ* is uniquely determined by the assumption that the spectral distribution continues to be Boltzmann like in the experimentally not resolved wavelength regime *λ*<550 nm, containing the ≈5% most energetic photons of the thermal cloud. The latter sets a value of *κ*=*U*/*U*(*λ*>550 nm)*≈*1.19, with *U*(*λ>*550 nm), denoting the energy contribution of photons with wavelength *λ>*550 nm.

**Figure 3:**
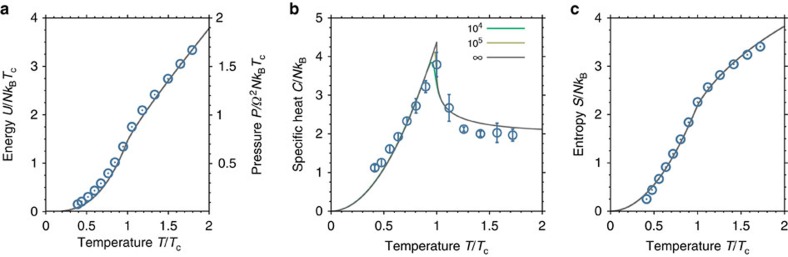
Caloric and entropic properties. (**a**) Internal energy (per photon) *U* normalized to the characteristic energy *k*_B_*T*_c_ as a function of the reduced temperature *T*/*T*_c_. In the classical high-temperature regime, energy scales linearly with temperature as is expected from Maxwell–Boltzmann-like statistics. The formation of the condensate is signalled by a change in slope close to *T=T*_c_. For an ideal two-dimensional trapped Bose gas, which is realized in our experiment in good approximation, the internal energy of the photon gas can moreover be linked to its pressure *P* by *P*=(1/2)Ω^2^*U*, see axis on the right-hand side and the main text for details (Ω is the trapping frequency). Experiments are carried out at constant temperature *T*=300 K and photon numbers ranging from *N*≈30,000 to *N*≈550,000, whereby an increase of the photon number corresponds to a decrease in the critical temperature *T*_c_=*T*_c_(*N*) of the system. s.e.m.'s are smaller than the point size. (**b**) Specific heat (per photon) versus the reduced temperature *T*/*T*_c_, showing a cusp singularity at criticality (circles). In the high-temperature (classical) regime, the specific heat reaches a limiting value of 2*k*_B_ per photon. At criticality *T*=*T*_c_, the heat capacity reaches a maximum value of *C*(*T*_c_)/*N*=(3.8±0.3)*k*_B_. The solid lines give the specific heat of the two-dimensional harmonically trapped ideal Bose gas for varying total particle numbers. The error bars indicate s.e.m. (**c**) Entropy per photon as a function of the reduced temperature *T*/*T*_c_ (circles), along with the theoretically expected ideal Bose gas behaviour (solid line). The data are derived from a numerical integration of the specific heat curve of **b** (see text for details). The entropy monotonically decreases for decreasing temperatures, reaching a minimum value of ≈0.2*k*_B_ per photon at the lowest achieved temperature. s.e.m.'s are smaller than the point size.

In Fig. 3a, the average energy *U*/*N* normalized to the characteristic energy at criticality *k*_B_*T*_c_ is plotted versus the reduced temperature *T/T*_c_, showing good agreement with the theoretical expectations for an ideal Bose gas (solid lines). At higher temperatures *T>T*_c_, the energy shows a linear scaling with temperature, as expected in the classical limit, where Maxwell–Boltzmann statistics applies. In the vicinity of the condensation threshold, the energy curve changes slope, as is more clearly revealed in the graph of the heat capacity in Fig. 3b. The data points are obtained by numerically differentiating the measured energy curve with respect to temperature, following *C*=∂*U*/∂*T*=*k*_B_ ∂(*U*/*k*_B_*T*_c_)/∂(*T*/*T*_c_). The obtained heat capacity data (circles) shows the characteristic λ-like shape as predicted theoretically (solid lines). In the high-temperature (classical) regime, *C* reaches a limiting value of 2*k*_B_ per photon. The latter stems from the four degrees of freedom, two kinetic (*k*_*x*_ and *k*_*y*_) and two potential (*x* and *y*), which quadratically enter the photon energy of equation (1) and each contribute with *k*_B_/2 to the specific heat (equipartition theorem). At criticality, the heat capacity shows a cusp with a maximum value of *C*(*T*_c_)/*N*=(3.8±0.3)*k*_B_, slightly below the theoretically expected value in the thermodynamic limit of 6*ζ*(3)/*ζ*(2)*k*_B_≈4.38*k*_B_ (refs 22, 23, 24). Most of this discrepancy can be explained by the finite size effects. From an exact numerical evaluation of the Bose–Einstein distribution function, one can obtain the specific heat for finite system sizes (solid lines in Fig. 3b). For the given photon numbers near *N*≈90,000 at criticality, the finite size effects reduce the specific heat maximum to a value of 4.11*k*_B_, which agrees with the measured value within the experimental uncertainties. For *T<T*_c_, the heat capacity monotonically decreases for smaller temperatures, dropping below the classical value of 2*k*_B_ per photon at *T/T*_c_≈0.7, and being consistent with reaching zero at *T*=0, as would be demanded by the third law of thermodynamics. Presently, the minimum achieved temperature is *T/T*_c_≈0.4, corresponding to condensate fractions of up to 84%, due to limited available pump power.

The specific heat furthermore gives access to other thermodynamic quantities, as the entropy, which can be determined by the integral *S*(*T*)=∫_*0*_^*T*^*C*(*T*′)/*T*′ *dT*′, see Fig. 3c for the corresponding data. As the specific heat *C*(*T*) is not known for temperatures below *T/T*_c_≈0.4, there is one free parameter, the constant offset *S*(*T/T*_c_≈0.4) that needs to be set to evaluate the integral and to match the experimental data (circles) to the theoretical prediction (solid line). The entropy curve monotonically decreases with decreasing temperature, reaching a minimum value of ≈0.2*k*_B_ per photon for the lowest obtained temperature. Although we presently cannot access temperatures closer to zero, the observed drop-off of the entropy curve towards lower temperatures is in accordance with the third law of thermodynamics. Note that the entropy per particle (as a function of chemical potential) also has been experimentally determined for a trapped two-dimensional atomic Bose gas, reaching entropies as low as 0.06(1)*k*_B_ (ref. 29).

## Discussion

For a gas of non-interacting bosons, which in good approximation is realized in our experiment as demonstrated by the results given in Figs 2 and 3, one can readily link the internal energy of the gas to its pressure. In general, in the presence of a trapping potential, the pressure of a gas becomes position dependent and thus cannot serve as a global thermodynamic variable. To account for this, it has been proposed to use two global conjugated variables, harmonic volume *V* and harmonic pressure *P*, respectively^11^,^30^. For a harmonically trapped two-dimensional gas, the harmonic volume is defined as *V*=Ω^−2^, with Ω as the trapping frequency. This quantity does not have the physical units of a volume, however, it shares the same scaling with the trapping geometry as the ‘true' volume of the confined gas. The harmonic pressure then follows via the usual thermodynamic relation *P*=−∂*φ*_G_/∂*V*, where *φ*_G_ is the grand potential. For a two-dimensional trapped gas of non-interacting bosons, the pressure can be shown to be related to the internal energy via *P*=(1/2)Ω^2^*U*. Thus, Fig. 3a here does not only describe the energy as a function of temperature *U*=*U*(*T/T*_c_) (caloric equation of state) but also delivers the pressure dependence *P*=*P*(*T/T*_c_) (thermal equation of state) for a given harmonic volume *V* or trapping frequency Ω.

To conclude, we have determined calorimetric properties of a Bose–Einstein-condensed photon gas, in particular the temperature dependence of energy, heat capacity and entropy. Critical behaviour of the photon gas is clearly demonstrated by a cusp in the specific heat curve at the condensation threshold. For the chosen experimental conditions, for example, sufficient photon reabsorption and nearly homogenous pump geometry, we do not observe significant deviations from the theoretically expected behaviour of a fully equilibrated Bose gas. In comparison with other systems exhibiting Bose–Einstein condensation, the here-investigated photon gas comes closest to an ideal, that is, interaction-free, gas of bosons, allowing to match experimental results with precise, and even exact, theoretical predictions. In particular, we find that the experimentally determined specific heat of the photon gas agrees with the quantum statistical predictions down to the level of finite size effects. For the future, the spectroscopic calorimetry of a quantum-degenerate photon gas could lead to new experimental schemes for precision measurements of thermodynamic quantities as the Boltzmann constant^31^,^32^.

## Methods

### Microcavity set-up

In our experiment, photons are stored inside a microcavity build up from two gyro-quality mirrors. The dielectric, spherically shaped mirrors have a reflectivity in excess of *r*≈0.99997 in the relevant wavelength region of this experiment (*λ*=530–590 nm), providing a cavity finesse of order of *F*≈10^5^ for the empty cavity. Both the mirrors share the same radius of curvature of *R*=1 m and are typically separated by *D*_0_≈1.7 μm, corresponding to a free spectral range of order Δ*λ*_FSR_≈100 nm, which is comparable to the spectral width of the dye emission. In this situation, the dye emission is restricted to cavity modes with the longitudinal wave number *q*=8, effectively reducing the thermalization dynamics of the photon gas to the remaining two transversal mode numbers. The effective mass and trapping frequency introduced in equation (1) depend on the cavity geometry and typically take values of *m*≈6.7 × 10^−36^ kg and Ω≈2*π*·36.5 GHz in our experiment. The cavity geometry is stabilized passively, by mechanical contact of the two mirrors that strongly damps fast mechanical oscillations, as well as actively utilizing a piezo translation stage that counteracts long time drifts of the resonance.

As a heat bath for the photon gas, we use a filtered solution of 10^−3^ mol l^−1^ rhodamine 6G ethylene glycol (fluorescence quantum yield *η*≈0.95, index of refraction of the solvent *n*=1.43). This dye fulfils the Kennard–Stepanov law *B*_21_(*ω*)/*B*_12_(*ω*)≈exp(−(*ω*−*ω*_ZPL_)/*k*_B_*T*), relating the Einstein coefficients of absorption *B*_12_(*ω*) and emission *B*_21_(*ω*) at a given frequency *ω* to the Boltzmann factor of that frequency (*ω*_ZPL_ is the zero-phonon line of the dye), which is essential for the light–matter thermalization process. The optical medium is spatially homogeneously pumped by a spectrally off-resonant laser system at a wavelength of *λ*_exc_=532 nm under an angle of ≈45° with respect to the optical axis exploiting the first reflectivity minimum at higher angles of incidence. To avoid excess population of long-lived dye triplet states and photobleaching two AOM are used to chop the pump light to pulses of 400 ns length with a repetition rate of 400 Hz.

## Additional information

**How to cite this article:** Damm, T. *et al*. Calorimetry of a Bose–Einstein-condensed photon gas. *Nat. Commun.* 7:11340 doi: 10.1038/ncomms11340 (2016).

## References

[b1] Tilley D. R. & Tilley J. Superfluidity and Superconductivity CRC Press (1990).

[b2] Lipa J. A., Nissen J. A., Stricker D. A., Swanson D. R. & Chui T. C. P. Specific heat of liquid helium in zero gravity very near the lambda point. Phys. Rev. B 68, 174518 (2003).

[b3] London F. The λ-phenomenon of liquid helium and the Bose-Einstein degeneracy. Nature 141, 643–644 (1938).

[b4] Einstein A. Quantum theory of the monoatomic ideal gas, part II. Sitzber. Preuss. Akad. 1, 3–14 (1925).

[b5] Penrose O. & Onsager L. Bose-Einstein condensation and liquid helium. Phys. Rev. 104, 576–584 (1956).

[b6] Anderson M. H., Ensher J. R., Matthews M. R., Wieman C. E. & Cornell E. A. Observation of Bose-Einstein condensation in a dilute atomic vapor. Science 269, 198–201 (1995). 17789847 10.1126/science.269.5221.198

[b7] Davis K. B. . Bose-Einstein condensation in a gas of sodium atoms. Phys. Rev. Lett. 75, 3969–3973 (1995). 10059782 10.1103/PhysRevLett.75.3969

[b8] Ku M. J. H., Sommer A. T., Cheuk L. W. & Zwierlein M. Revealing the superfluid lambda transition in the universal thermodynamics of a unitary Fermi gas. Science 335, 563–567 (2012). 22245739 10.1126/science.1214987

[b9] Blakie P. B., Toth E. & Davis M. J. Calorimetry of Bose-Einstein condensates. J. Phys. B: At. Mol. Opt. Phys. 40, 3273–3282 (2007).

[b10] Ensher J. R., Jin D. S., Matthews M. R., Wieman C. E. & Cornell E. A. Bose-Einstein condensation in a dilute gas: measurement of energy and ground-state occupation. Phys. Rev. Lett. 77, 4984–4987 (1996). 10062686 10.1103/PhysRevLett.77.4984

[b11] Shiozaki R. F. . Measuring the heat capacity in a Bose-Einstein condensation using global variables. Phys. Rev. A 90, 043640 (2014).

[b12] Kasprzak J. . Bose-Einstein condensation of exciton polaritons. Nature 443, 409–414 (2006). 17006506 10.1038/nature05131

[b13] Balili R., Hartwell V., Snoke D., Pfeiffer L. & West K. Bose-Einstein condensation of microcavity polaritons in a trap. Science 316, 1007–1010 (2007). 17510360 10.1126/science.1140990

[b14] Demokritov S. O. . Bose-Einstein condensation of quasi-equilibrium magnons at room temperature under pumping. Nature 443, 430–433 (2006). 17006509 10.1038/nature05117

[b15] Klaers J., Schmitt J., Vewinger F. & Weitz M. Bose-Einstein condensation of photons in an optical microcavity. Nature 468, 545–548 (2010). 21107426 10.1038/nature09567

[b16] Klaers J., Vewinger F. & Weitz M. Thermalization of a two-dimensional photonic gas in a ‘white wall' photon box. Nat. Phys. 6, 512–515 (2010).

[b17] Schmitt J. . Observation of grand-canonical number statistics in a photon Bose-Einstein condensate. Phys. Rev. Lett. 112, 030401 (2014). 24484122 10.1103/PhysRevLett.112.030401

[b18] Klaers J., Schmitt J., Damm T., Vewinger F. & Weitz M. Statistical physics of Bose-Einstein-condensed light in a dye microcavity. Phys. Rev. Lett. 108, 160403 (2012). 22680703 10.1103/PhysRevLett.108.160403

[b19] Nyman R. A. & Szymanska M. H. Interactions in dye-microcavity photon condensates and the prospects for their observation. Phys. Rev. A 89, 033844 (2014).

[b20] Kirton P. & Keeling J. Nonequilibrium model of photon condensation. Phys. Rev. Lett. 111, 100404 (2013). 25166637 10.1103/PhysRevLett.111.100404

[b21] de Leeuw A.-W., Stoof H. T. C. & Duine R. A. Schwinger-Keldysh theory for Bose-Einstein condensation of photons in a dye-filled optical microcavity. Phys. Rev. A 88, 033829 (2013).

[b22] Haugset T., Haugerud H. & Andersen J. O. Bose-Einstein condensation in anisotropic harmonic traps. Phys. Rev. A 55, 2922–2929 (1997).

[b23] Klünder B. & Pelster A. Systematic semiclassical expansion for harmonically trapped ideal Bose gases. Eur. Phys. J. B 68, 457–465 (2009).

[b24] Kozhevnikov A. A. Bose gas in power-like spherically symmetric potential in arbitrary spatial dimensionality. Acta Phys. Pol. B 43, 2089–2102 (2012).

[b25] Marelic J. & Nyman R. A. Experimental evidence for inhomogeneous pumping and energy-dependent effects in photon Bose-Einstein condensation. Phys. Rev. A 91, 033813 (2015).

[b26] Kirton P. & Keeling J. Thermalization and breakdown of thermalization in photon condensates. Phys. Rev. B 91, 033826 (2015).

[b27] Keeling J. & Kirton P. Spatial dynamics, thermalization, and gain clamping in a photon condensate. Phys. Rev. A 93, 013829 (2016).

[b28] Tammuz N. . Can a Bose gas be saturated? Phys. Rev. Lett. 106, 230401 (2011). 21770484 10.1103/PhysRevLett.106.230401

[b29] Yefsah T., Desbuquois R., Chomaz L., Günter K. J. & Dalibard J. Exploring the thermodynamics of a two-dimensional Bose gas. Phys. Rev. Lett. 107, 130401 (2011). 22026829 10.1103/PhysRevLett.107.130401

[b30] Romero-Rochin V. Equation of state of an interacting Bose gas confined by a harmonic trap: the role of the ‘harmonic' pressure. Phys. Rev. Lett. 94, 130601 (2005). 15903978 10.1103/PhysRevLett.94.130601

[b31] de Podesta M. . A low-uncertainty measurement of the Boltzmann constant. Metrologia 50, 354–376 (2013).

[b32] Castrillo A. . The Boltzmann constant from the shape of a molecular spectral line. J. Mol. Spectrosc. 300, 131–138 (2014).

